# Implementation and evaluation of a large-scale postpartum family planning program in Rwanda: study protocol for a clinic-randomized controlled trial

**DOI:** 10.1186/s13063-022-06261-5

**Published:** 2022-04-22

**Authors:** Kristin M. Wall, Rosine Ingabire, Amelia Mazzei, Claudine Umuhoza, Rachel Parker, Amanda Tichacek, Azhar Nizam, Jessica M. Sales, Lisa B. Haddad, Phaedra Corso, Susan Allen, Julien Nyombayire, Etienne Karita

**Affiliations:** 1 Department of Epidemiology, School of Public Health, Emory University, Atlanta, USA; 2 Projet San Francisco (PSF)/Center for Family Health Research (CFHR), Kigali, Rwanda; 3 Department of Pathology, School of Medicine, Emory University, Atlanta, USA; 4 Department of Biostatistics and Bioinformatics, School of Public Health, Emory University, Atlanta, USA; 5 Department of Behavioral, Social and Health Education Sciences, School of Public Health, Emory University, Atlanta, USA; 6 Population Council, New York, USA; 7 Office of Research, Department of Health Policy, Kennesaw State University, Kennesaw, USA

**Keywords:** Postpartum, Family planning, Contraception, Birth spacing, Implementation, Rwanda, Stepped-wedge randomized trial

## Abstract

**Background:**

Though the Rwandan Ministry of Health (MOH) prioritizes the scale-up of postpartum family planning (PPFP) programs, uptake and sustainability of PPFP services in Rwanda are low. Furthermore, highly effective long-acting reversible contraceptive method use (LARC), key in effective PPFP programs, is specifically low in Rwanda. We previously pilot tested a supply-demand intervention which significantly increased the use of postpartum LARC (PPLARC) in Rwandan government clinics. In this protocol, we use an implementation science framework to test whether our intervention is adaptable to large-scale implementation, cost-effective, and sustainable.

**Methods:**

In a type 2 effectiveness-implementation hybrid study, we will evaluate the impact of our PPFP intervention on postpartum LARC (PPLARC) uptake in a clinic-randomized trial in 12 high-volume health facilities in Kigali, Rwanda. We will evaluate this hybrid study using the RE-AIM framework. The independent effectiveness of each PPFP demand creation strategy on PPLARC uptake among antenatal clinic attendees who later deliver in a study facility will be estimated. To assess sustainability, we will assess the intervention adoption, implementation, and maintenance. Finally, we will evaluate intervention cost-effectiveness and develop a national costed implementation plan.

**Discussion:**

Adaptability and sustainability within government facilities are critical aspects of our proposal, and the MOH and other local stakeholders will be engaged from the outset. We expect to deliver PPFP counseling to over 21,000 women/couples during the project period. We hypothesize that the intervention will significantly increase the number of stakeholders engaged, PPFP providers and promoters trained, couples/clients receiving information about PPFP, and PPLARC uptake comparing intervention versus standard of care. We expect PPFP client satisfaction will be high. Finally, we also hypothesize that the intervention will be cost-saving relative to the standard of care. This intervention could dramatically reduce unintended pregnancy and abortion, as well as improve maternal and newborn health. Our PPFP implementation model is designed to be replicable and expandable to other countries in the region which similarly have a high unmet need for PPFP.

**Trial registration:**

ClinicalTrials.gov NCT05056545. Registered on 31 March 2022.

## Administrative information

Note: The numbers in curly brackets in this protocol refer to SPIRIT checklist item numbers. The order of the items has been modified to group similar items (see https://www.equator-network.org/reporting-guidelines/spirit-2727-statement-defining-standard-protocol-items-for-clinical-trials/).

**Table:** 

Title {1}	Implementation and evaluation of a large-scale postpartum family planning program in Rwanda: study protocol for a cluster-randomized controlled trial
Trial registration {2a and 2b}.	Clinical ClinicalTrials.gov ID: NCT05056545
Protocol version {3}	12Feb21 v1.0
Funding {4}	National Institutes of Health
Author details {5a}	Kristin M. Wall, Department of Epidemiology, School of Public Health, Emory University Rosine Ingabire, PSF/CFHR, Kigali, Rwanda Amelia Mazzei, Department of Pathology, School of Medicine, Emory University; PSF/CFHR, Kigali, Rwanda Claudine Umuhoza, PSF/CFHR, Kigali, Rwanda Amanda Tichacek, Department of Pathology, School of Medicine, Emory University Azhar Nizam, Department of Biostatistics and Bioinformatics, School of Public Health, Emory University Jessica M. Sales, Department of Behavioral, Social and Health Education Sciences, School of Public Health, Emory University Lisa B. Haddad, Population Council Phaedra Corso, Kennesaw State Susan Allen, Department of Pathology, School of Medicine, Emory University Rachel Parker, Department of Pathology, School of Medicine, Emory University Etienne Karita, Department of Pathology, School of Medicine, Emory University; PSF/CFHR, Kigali, Rwanda
Name and contact information for the trial sponsor {5b}	National Institutes of Health
Role of sponsor {5c}	The National Institutes of Health had no role in the study design; collection, management, analysis, or interpretation of data; writing of the report; or the decision to submit the report for publication.

## Introduction

### Background and rationale {6a}

Uptake and sustainability of postpartum family planning (PPFP) services in Rwanda are low [1]. The Rwandan Demographic Health Survey (DHS) estimates that 51% of women have an unmet need for PPFP, which is higher than the unmet need during non-postpartum periods [2]. Long-acting reversible contraceptives (LARCs), including the copper intrauterine device (IUD) and hormonal contraceptive implant, are fundamental to PPFP programs. LARCs are highly effective and are the only reversible methods that may be safely used in the early postpartum period by breastfeeding women. As in most countries, postpartum use of long-acting reversible contraceptives (PPLARC) is low in Rwanda: among contracepting postpartum women, 8% use contraceptive implants and < 1% use the intrauterine device (IUD) [3, 4].

To address the high unmet need for PPFP and PPLARC in Rwanda, in 2017–2018, our research group worked closely with the Rwanda Ministry of Health (MOH) to develop and pilot test a theory-based, multi-level intervention targeting PPFP supply and demand in five government health facilities in Kigali, the capital. We trained government clinic providers to promote and provide PPFP methods, and we developed an educational counseling flipchart which detailed all PPFP options (PPLARC, injectables, pills, etc.) which was provided to 12,068 pregnant and postpartum women/couples in health facilities and the surrounding community. During our pilot, LARC uptake up to 6 weeks after delivery increased significantly (with 3372 PPIUD inserted representing a 2710% increase compared to the 6 months prior to our pilot, and 1252 implants inserted representing a 178% increase compared to the 6 months prior to our pilot), PPFP feasibility and acceptability were high among providers and clients, and side-effects were rare [5, 6].

While our short pilot was highly impactful in increasing PPFP uptake, much remains unknown: Is our PPFP intervention scalable without sacrificing quality? Will the inclusion of husbands, 85% of whom attend the first antenatal visit with their wives, increase PPLARC acceptability and uptake? Does the use of a Happy Client model of peer testimonial or PPFP promotions delivered by community health workers (CHW) increase intervention effectiveness? Is the intervention cost-effective? What demand creation strategies are most cost-effective? Is the intervention sustainable?

We now will conduct a type 2 effectiveness-implementation hybrid study [7, 8] to test the hypothesis that our PPFP intervention is adaptable to large-scale implementation, cost-effective, and sustainable. This intervention could dramatically reduce unintended pregnancy and abortion and improve birth spacing and maternal and newborn health in Rwanda.

### Objectives {7}

Aim 1: Implement the intervention in 12 high-volume health facilities: Guided by the Consolidated Framework for Implementation Research [9, 10], we will engage stakeholders and assess facility organizational readiness prior to implementation. No PPFP activities are currently taking place in these facilities. We will implement the PPFP intervention in a clinic-randomized stepped-wedge design. We expect to deliver PPFP promotional counseling to over 21,000 pregnant or postpartum women/couples.

Aim 2: Evaluate intervention effectiveness and implementation: The RE-AIM framework [11] will guide the outcome evaluation. We hypothesize that the intervention will significantly increase the number of stakeholders engaged, PPFP providers and promoters, couples/clients receiving information about PPFP, and LARC uptake comparing intervention versus standard of care. We expect PPFP client satisfaction will be high, and side effects will be rare. The independent effectiveness of each demand creation strategy on LARC uptake will be estimated. We assess the measures of intervention adoption, implementation, and maintenance at the patient, provider, and stakeholder levels to assess the intervention sustainability.

Aim 3: Evaluate intervention cost-effectiveness and cost-utility: We employ standard methods [12, 13] to estimate the costs and incremental cost-effectiveness and cost-utility of the intervention relative to the standard of care from the societal perspective. The cost-effectiveness of each demand creation strategy will be estimated. We hypothesize that the intervention will be cost-saving relative to the standard of care. A costed implementation plan will be developed to guide Rwandan MOH decision-making for the nationwide roll-out of PPFP services.

### Trial design {8}

We will implement the intervention in 12 facilities in a clinic-randomized stepped-wedge design. The stepped-wedge design will ensure that each facility receives the intervention over time and provides its own standard of care control, limiting temporal bias. Facilities are high-volume and have similar client volume and staffing-to-patient ratios. Each clinic will be randomized using the SAS random number generator (SAS Institute v9.4, Cary, NC). The allocation ratio will be 1:1. This is a superiority trial.

## Methods: participants, interventions, and outcomes

### Study setting {9}

The Projet San Francisco (PSF)/Center for Family Health Research (CFHR) is an accredited non-profit organization established in Kigali, Rwanda, in 1986 and is committed to the pursuit of excellence in research and service delivery to improve family planning and LARC. PSF/CHFR will oversee all in-country implementation activities.

We will implement the PPFP intervention in 12 intervention facilities with high-volume labor and delivery (L&D) departments and will use our 5 pilot facilities as training sites in Kigali. These 17 facilities include 15 health centers (which provide antenatal care [ANC], family planning, and infant vaccination [IV] and refer L&D cases to adjoining hospitals) and 2 stand-alone hospitals (which provide routine L&D services and also receive referrals of high-risk and complex obstetric cases from health centers). The selected facilities are currently staffed with roughly 443 facility-affiliated CHWs, 35 ANC nurses, 188 L&D nurses/midwives, 41 family planning nurses, and 41 IV nurses. Additionally, over a 3-month period, these facilities serve roughly 4657 ANC clients, 4196 L&D clients, and 4371 IV clients. All facilities have the infrastructure for PPFP provision and procurement; though no formal PPFP initiatives are currently taking place, PP implant insertions are infrequent, and no providers are trained to insert PPIUDs. Our team has worked in these clinics (conducting clinic logbook data abstraction, implementing couples’ HIV counseling and testing, implementing interval LARC services, and surveying providers and patients) for over 10 years [6, 14–21].

### Eligibility criteria {10}

Aim 1: Provider and stakeholder in-depth interviews

All participants will be government employees of at least 18 years of age who provide written informed consent to participate in in-depth interviews.

Aim 2: Implement PPFP services in a cluster-randomized stepped-wedge trial

Eligible PPFP providers will be women working in L&D or family planning at one of the study facilities. Eligible Happy Client promotors will be postpartum women who received promotions and selected a PPFP method. Eligible nurse and CHW promotors will be women working as nurses or CHWs at one of the intervention facilities. All women eligible for PPFP services in this study will be at any stage of pregnancy or up to 14 weeks postpartum and receiving ANC, L&D, IV, or postpartum services at one of the intervention facilities.

Aim 3: Client/couple, provider, and stakeholder in-depth interviews

All participants will be at least 18 years of age who provide written informed consent to participate in the in-depth interviews. Clients/couples will be those who received PPFP counseling. Providers and stakeholders will be government employees.

### Who will take informed consent? {26a}

Informed consent will be obtained from in-depth interview participants by trained study staff prior to the interview. Aim 1 interviews will be held during year 1 of the study, while the aim 2 interviews will take place 9 months after receiving PPFP promotions for PPFP clients and at the end of year 4/beginning of year 5 for stakeholder and provider interviews.

### Additional consent provisions for collection and use of participant data and biological specimens {26b}

This protocol received a waiver of consent for service delivery activities from the Emory and Rwandan Institutional Review Boards (IRBs). This trial does not involve collecting biological specimens.

## Interventions

### Explanation for the choice of comparators {6b}

A standard of care control was selected since it is the current alternative to offering the PPFP intervention. The stepped-wedge design will ensure that each facility receives the intervention over time and provides its own standard of care control. Facilities are high-volume and have similar client volume and staffing-to-patient ratios.

### Intervention description {11a}

We are evaluating a supply-demand intervention in a clinic-randomized design. The trial methods are provided here.

#### Organizational readiness checklist

Guided by the Consolidated Framework for Implementation Research (CFIR) [9, 10], we will conduct facility needs assessments to understand the readiness and barriers to intervention implementation. Checklists will measure facility volume and staffing, pharmacy capacity, physical space for the PPFP intervention, current contraception workflows and procurement systems, prior PPFP trainings, PPFP stocks, and any procedures (i.e., PPFP counseling tools or data collection systems) supporting PPFP supply or demand. Each assessment will take 1 h and will be conducted by trained research staff.

#### In-depth interviews

To explore the barriers to intervention implementation, we will conduct one-on-one in-depth interviews with *n* = 12 facility directors, *n* = 24 nurses in charge of L&D and family planning (2/facility), *n* = 24 CHW (2/facility), and *n* = 5 MOH stakeholders. CFIR provides guidance for qualitative semi-structured interview constructs [10]. Interviews will take 30 min, and participants will be compensated. Interview guides will be translated into the primary local language (Kinyarwanda) and pre-tested. Interviews will be anonymized and conducted by the research staff experienced in leading in-depth interviews.

#### PPFP service delivery training

The 5 pilot facilities (which are not randomized) will serve as sites to train and mentor nurses and midwives working in L&D and family planning at the 12 intervention facilities. The training will include a 2-day didactic session covering PPFP provision, follow-up, and use of the PPFP counseling flipchart to promote all PPFP method options (PPLARC, injectables, pills, etc.); mock counseling sessions; and PPIUD and PP implant insertion and removal trainings. Pre/post-training tests will consist of 10 true/false questions. Re-training will be offered after 1 week for trainees who fail to score > 80% on the post-training evaluation. After passing the didactic training, nurses and midwives who will be charged with insertions will be required to correctly insert 5 PPIUDs and 5 PP implants under supervision to be PPFP certified. The PPIUD insertions must include at least one of each of the following timings (which require different insertion techniques): immediate post-placental (within 10 min of placental delivery), 10 min to 48 h postpartum, and 4–14 weeks post-delivery.

#### PPFP demand creation training

We will train the government clinic staff in family planning, ANC, L&D, and IV to promote PPFP at the selected facilities. Trainings will comprise a 3-h-long didactic session led by the trained research staff followed by two supervised counseling sessions. CHWs from the health centers in charge of pregnant women and newborn health will receive a 1-day training on the PPFP flipchart. If interested in PPFP, women will be referred to the facility by their CHW. We will develop training materials to coach Happy Clients to discuss their own experiences with PPFP and address common concerns (as identified in our formative work and also addressed in the promotional flipchart). Happy Clients will be distributed equally across intervention facilities, affiliated with only one facility, and will be partnered with trained PPFP counselors. Finally, we will create an educational video in which a nurse counselor describes the PPFP flipchart information, visually shows each PPFP option, and men and Happy Clients give brief testimonies as to their experiences with different PPFP methods. Videos providing PPFP information will be shown in the ANC, L&D, and IV waiting areas in intervention facilities.

#### Stakeholder engagement

We will regularly engage stakeholders by presenting interim findings at quarterly Family Planning Technical Working Group meetings.

#### Follow-up procedures

PPFP follow-up appointments will be scheduled 6 weeks after insertion to correspond with the first IV visit. Women will be asked to self-report the side effects including signs of infection, and for PPIUD users, IUD string placement will be checked via bimanual pelvic exam. IUD strings will be trimmed as needed and a pelvic ultrasound given if the strings are not visible during physical exam. Women may decide to discontinue or request a new family planning method of their choice at any time.

#### Reimbursements

Using the performance-based financing (PBF) system used by the Rwanda Ministry of Health as a guide [22], providers will be reimbursed for PPFP methods inserted, and these payments will be made to their facility and included in addition to their regular PBF pay. For context, providers receive PBF pay of US $0.60/new contraceptive method user regardless of method type, and the average salary for nurses working in family planning or L&D is US $124–364/month, depending on the educational level. CHW and Happy Clients will be reimbursed for their work.

### Criteria for discontinuing or modifying allocated interventions {11b}

There will be no special criteria for discontinuing or modifying allocated interventions.

### Strategies to improve adherence to interventions {11c}

We will re-train providers in relevant service provision and promotional activities as needed.

### Relevant concomitant care permitted or prohibited during the trial {11d}

All side effects or incidental findings (for example, from any needed pelvic or ultrasound exam) will be shared with participants and their government health facility provider so that standard care treatments may be provided by the health facility.

### Provisions for post-trial care {30}

All clinical care including management of PPFP side effects will be provided by government facility personnel at health centers as part of usual care at no cost to the patient or health facility.

### Outcomes {12}

RE-AIM will guide outcome assessment (Table 1).

**Table 1:** RE-AIM domains and measures

Domains	Outcome measures	Data source
**Reach**	^a^Stakeholders engaged and PPFP providers/promoters trained	Advocacy and training study log
	^a^Couples/clients receiving information about PPFP	PPFP logbook
**Effectiveness**	^a^Clients receiving PPFP/PP LARC methods PPFP side effects	PPFP logbook
**Adoption**	^a^Providers/promoters providing PPFP	Advocacy and training study log
**Implementation**	Provider/promoter time to provide PPFP Patient wait times to receive PPFP Implementation fidelity	Time motion studies, fidelity checklist
**Maintenance**	Woman/couple-level: continued PPFP use at 9 months, factors associated with PPFP uptake and continued use	Interviews, PPFP logbook
	Provider/facility-level: progress toward institutionalizing PPFP, factors associated with PPFP continued provision at the end of implementation	Interviews, standardized checklist
	Stakeholder-level: progress of the Ministry of Health to nationalize PPFP services at the end of implementation	Interviews

^a^Quantitative outcomes used in power analyses, see Table 5

#### Reach and adoption

Data on the number and proportion of stakeholders engaged, providers trained, and promoters trained will be abstracted from the advocacy and training study logs. Data on the number and proportion of couples/clients receiving information about PPFP will be abstracted from PPFP logbooks.

#### Effectiveness

Data on the number and proportion of clients receiving PPFP/PPLARC methods and experience of side effects will be abstracted from PPFP logbooks.

#### Implementation

We will conduct time-motion studies wherein the program coordinator will follow a random 5% of women/couples offered the intervention through their flow of clinical care to record provider/promoter time, patient wait times, and regular clinical operation disruptions. We expect that < 20 min/day will be required by nurses/counselors to deliver the PPFP intervention. During these time-motions studies, we will also assess the implementation fidelity defined using the empirically tested Conceptual Framework for Implementation Fidelity [23, 24] as the proportion of providers adhering to the intervention protocol content as assessed using a standardized checklist of key implementation steps. We expect that the intervention will be implemented per protocol in 90% of observations. The time-motion studies, inclusive of fidelity assessments, will be conducted at month 2 and at 6 months after implementation with re-training as necessary.

#### Maintenance

##### Woman/couple level

We will abstract clinic logbook data to calculate the proportion of women who are still using the PPFP method 9 months after uptake. We will conduct individual in-depth interviews with PPFP users and non-users who received the intervention and their male partners 9 months after receiving promotions. Participants identified from PPFP logbooks will be interviewed at the clinic to gain insight into women’s and men’s experiences regarding PPFP side effects, reasons for uptake/continued use/non-use, service delivery environment, demand creation strategies, and PPFP methods.

##### Provider level

Drawing from the diffusion of innovation theory [25], we will conduct in-depth interviews with PPFP trainees, CHW, Happy Clients, and all facility directors at the end of the implementation. Interview questions will include perceptions of the degree to which intervention is better than SOC; perceptions about how consistent intervention implementation and the demand creation strategies are with the values, culture, experiences, and needs of pregnant women and partners; the perception that intervention implementation is needed; general perceptions of difficulty of intervention implementation and the demand creation strategy burden/cost on providers or stakeholders; evaluation of facilitators/barriers and suggestions for improvement in intervention implementation (training and service delivery) for future refinement of the implementation strategies; and the extent to which providers and stakeholder feel that the intervention implementation improves health for women and families. During interviews, we will also use a standardized checklist to document ongoing or planned PPFP trainings at the facilities, PPFP promotional activities, continued use of PPFP logbooks, discussion of PPFP at regular staff meetings, and pharmacy coordination with providers to ensure PPFP supplies/stocks.

##### Stakeholder level

We will conduct in-depth interviews among stakeholders including MOH and Rwandan Family Planning Technical Working Group members, clinic directors, and nurse administrators. Interviews will take place at the end of the intervention period and will assess the continued engagement, buy-in, and support for PPFP. To assess the national-level maintenance, we will also assess the progress of the MOH to institutionalize PPFP services via renewed public support/statements, allocation of training funds, or dedicated funding for PPFP.

#### Safety endpoints

PPFP side effects include pelvic pain, heavy menstrual bleeding/menorrhagia, dysmenorrhea, infection, perforation of the uterine wall, pelvic inflammatory disease (PID), headaches, irregular periods, nausea, blood clot, minor bleeding, severe bleeding, and pain.

### Participant timeline {13}

The timeline in Table 2 indicates the schedule of interviews and intervention implementation. Women will participate for a maximum of 54 weeks (40 weeks gestation plus 14 weeks postpartum), and in-depth interviews with women will occur 9 months after PPFP promotions. The timeline for implementation of the cluster-randomized stepped-wedge is shown in Table 3.

**Table 2:** Overall timeline of study activities by year (Y) (in 6-month intervals)

Aim	Study activity	Y1-1	Y1-2	Y2-1	Y2-2	Y3-1	Y3-2	Y4-1	Y4-2	Y5-1	Y5-2
1	Protocol development and IRB approvals	*X*									
	In-depth interviews		*X*								
	Facility needs assessments			*X*							
	Implement the intervention in a randomized stepped-wedge design and collect effectiveness and implementation data			*X*	*X*	*X*	*X*	*X*			
2	Evaluate intervention reach, effectiveness, and adoption								*X*	*X*	*X*
	Evaluate the intervention implementation (time-motion studies)			*X*	*X*	*X*	*X*	*X*			
	Evaluate intervention maintenance: women/couples			*X*	*X*	*X*	*X*	*X*			
	Evaluate the intervention maintenance: providers and stakeholders								*X*	*X*	
3	Develop costing tools	*X*	*X*								
	Collect cost data			*X*	*X*	*X*	*X*	*X*			
	Cost-effectiveness analyses and development of costed implantation plan								*X*	*X*	*X*
	Publications, dissemination								*X*	*X*	*X*

**Table 3:**
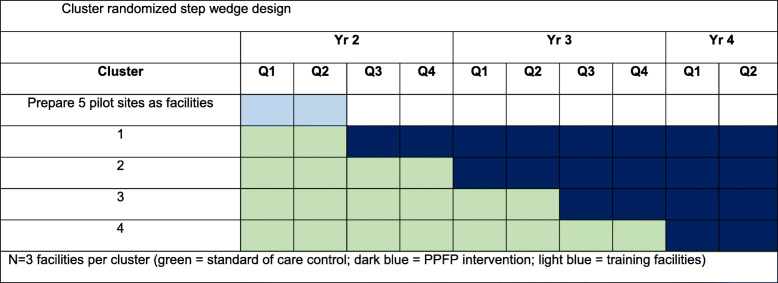
Cluster-randomized stepped-wedge design

### Sample size {14}

Table 4 shows the expected number of participants included in each study activity. We assume that the proposed sample sizes shown below for in-depth interviews will allow us to reach saturation. Based on facility volume and preliminary findings, we conservatively expect to deliver PPFP promotions to 12,714 in the 12 intervention facilities (18% of reproductive age women in the catchment area) and 21,383 women/couples total across the 17 facilities (12 intervention plus 5 training facilities).

**Table 4:** Expected number of participants included for each study activity

Specific aim	Activity	Total participants
Aim 1	In-depth interviews	12 facility directors 24 nurses in-charge of L&D and family planning 24 CHWs 5 MOH stakeholders
Aim 2	Intervention trainings	98 government facility PPFP promoters 68 community health worker promoters 78 government facility PPFP providers (70 certified) 36 Happy Client promoters
	Intervention recipients	We expect to deliver PPFP promotions to *n* = 12,714 women/couples
	In-depth interviews	25 women/couples 25 providers 25 stakeholders

We assume based on our pilot that promotions will reach 75% of pregnant women in health centers. Also extrapolating from our pilot data, we also expect to insert 3687 PPIUDs and 1399 PP implants in the 12 intervention facilities (6201 PPIUDs and 2352 PP implants total across the 17 facilities) (Table 5). Given the facility volume and previous experience, PPFP service delivery to this number of women is highly feasible. Sample size and power calculations account for intra-cluster correlation (ICC) which considers the between-cluster correlation, within-cluster correlation, and repeated measures. We assume pooled between- and within-cluster ICCs and a homogeneous intervention effect over time. We have > 80% power to detect all differences in the expected proportions shown in Table 5 comparing PPFP intervention to control.

**Table 5:** Expected deliverables during the study time period, *n* = 12 intervention facilities. Power to detect the differences between standard of care control and intervention: > 80%

Stakeholders	Control^**a**^			PPFP intervention		
	***N*** events	Denominator	Expected %	***N*** events	Denominator	Expected %^**b**^
Clinic directors engaged	2	12	17%	12	12	100%
**Promotions**						
Nurses^b^ trained to promote PPFP	7	215	3%	98	215	46%
CHWs trained to promote PPFP	0	313	0%	68	313	22%
**Providers at implementation facilities**						
Providers trained to provide PPFP	7	162	4%	78	162	48%
Providers certified to provide PPFP	0	162	0%	70	162	43%
**Clients delivering at implementation facilities**						
Promotions to pregnant or postpartum women/couples	268	17,870	2%	12,714	17,870	71%
PPIUD insertions	20	268	7%	3687	12,714	29%
PP implant insertions	14	268	5%	1399	12,714	10%

^a^Data from *n* = 12 government clinic logbooks, 2018

^b^Estimated from the pilot study [5]

^c^In ANC, L&D, FP, and IV

### Recruitment {15}

Aim 1/2: In-depth interviews: Participants will be recruited by random sample, purposively distributed across the intervention facilities.

Aim 2: Implement intervention services in a clinic-randomized stepped-wedge trial: All eligible nurse/midwives and CHW at the selected facilities will be recruited as PPFP providers and/or promoters. Happy Clients will be recruited by convenience, distributed across the intervention facilities. Women receiving intervention services will not be recruited; all women receiving care at one of the selected facilities during the intervention time period will be offered the intervention.

## Assignment of interventions: allocation

### Sequence generation {16a}

Each clinic will be randomized using the SAS random number generator (SAS Institute v9.4, Cary, NC). The allocation ratio will be 1:1. There will be no stratification or blocking. The facilities are all of similar, high-volume, and have similar client volume and staffing-to-patient ratios.

### Concealment mechanism {16b}

The allocation sequence will not be visible to the trial staff conducting randomization so that the assignment will be unknown to staff until each numbered, security-type envelope is opened.

### Implementation {16c}

The allocation sequence will be generated by trained study staff. Clinic intervention assignment will be conducted by trained study staff.

## Assignment of interventions: blinding

### Who will be blinded {17a}

There is no blinding in this study. Providers and clients in intervention facilities will know that the intervention is being implemented in that facility.

### Procedure for unblinding if needed {17b}

The design is open-label, so unblinding will not occur.

## Data collection and management

### Plans for assessment and collection of outcomes {18a}

The number of stakeholders engaged and providers trained and certified will be recorded in an Advocacy and Training logbook. CHW counseling activities will be measured on referral slips which will record if a male partner was present. Women/couples receiving one-on-one PPFP counseling will have their method of interest, whether a male partner was present, household income, and estimated date of delivery recorded in a PPFP logbook, developed by the study team during the pilot and maintained by government clinic staff. The PPFP logbook will also record PPFP uptake and, among PP LARC users, provider perception of ease of insertion, client anxiety during insertion, and client pain during insertion (scales of 1–10). During follow-up visits, the PPFP logbook will record PPFP side effects and method failures. Facility information, client’s age, and parity will be abstracted from government Family Planning logbooks.

### Plans to promote participant retention and complete follow-up {18b}

Aim 1/2: In-depth interviews: As these are one-time interviews, there is no retention plan.

Aim 2: Implement PPFP services in a clinic-randomized stepped-wedge trial: To increase attendance at PPFP follow-up appointments at one of the intervention facilities, we will reimburse women for their transportation/childcare costs for follow-up visits. To increase follow-up, we will also provide CHWs with lists of clients in their catchment areas who are pending follow-up to remind those women about their appointments.

Any identification of participation or follow-up rates that represent threats to our ability to sufficiently answer the research questions will be met with action by the PI to intervene to remedy the shortcomings.

### Data management {19}

PPFP logbooks will be maintained by trained PPFP providers in government facilities. Logbook data will be extracted into tablets weekly by the research field team through the mobile data collection platform Survey CTO v2.41 (Dobility, Cambridge, USA). Survey CTO data will be downloaded for weekly query and cleaning in a Microsoft Access database. Data will then be imported into SAS (Cary, NC) for analysis. A unique code will track clients from the community through ANC, L&D, and IV. This unique code is composed of personal information that the participant would remember (e.g., the first three letters of the mother’s name) but is unlinked to identifiers. We used this code in our previous pilot and were able to track > 95% of women who attended multiple visits at ANC, delivery/postpartum, and/or infant vaccination. For qualitative assessments, de-identified transcribed audio recordings will be stored on a secure server. Recruitment and missing data reports will be monitored weekly by the study coordinator, the PI, and the study statistician.

### Confidentiality {27}

We will minimize the risk of any loss of confidentiality throughout the study design and conduct. These features will be included in standard operating procedures (SOPs) as follows:

- All study procedures will be conducted in private, to the extent possible.
- A trained research staff member will administer all in-depth interviews and mixed-methods surveys conducted in specific aims 1 and 3, respectively.
- Individual subjects will not be identified by the data collected; it will be used for research purposes only and no personal identifiers will be used.
- All non-electronic research-related information will be securely stored in locked file cabinets in locked rooms with restricted access at PSF/CFHR.
- All electronic records will be kept in password-protected files. All electronic communications of study data will be through password-protected, encrypted files. All data storage at Emory University or PSF/CFHR will be on firewall-protected servers.
- All staff participating in data collection or management have been/will be certified in Good Clinical Practices (GCP) and will attend documented trainings regarding the protection of human subjects.
- Per the Data and Safety Monitoring Plan, data and data collection and management procedures will be reviewed at least annually.
- Informed consent documents will describe confidentiality and the legal limits of confidentiality.

No identifiable protected health information will be collected under this protocol. All data will be entered into a password-secured access database. Any non-electronic research-related information will be stored in locked file cabinets in locked rooms with restricted access.

### Plans for collection, laboratory evaluation, and storage of biological specimens for genetic or molecular analysis in this trial/future use {33}

This trial does not involve collecting biological specimens (please see Additional consent provisions for collection and use of participant data and biological specimens {26b}).

## Statistical methods

### Statistical methods for primary and secondary outcomes {20a}

#### Reach and adoption

We will tabulate, overall and by facility, the number of stakeholders engaged, providers trained, promoters trained, providers certified and pre/post-training evaluation scores, and clients who received one-on-one counseling. We hypothesize increases comparing intervention versus standard of care in stakeholders engaged, providers trained to promote and provide PPFP, and couples/clients receiving information about PPFP (Table 5). We expect 90% of trainees will receive certification to promote and provide PPFP.

#### Effectiveness

##### Primary analyses

We will tabulate, by facility, a total number of all PPFP methods provided and the proportion of PPFP uptake among women who delivered at one of our facilities (overall and by method type). PP LARC uptake (by method type) will be evaluated using logistic regression and general estimating equation methods to account for the clinic-randomized design. The main predictor will be the intervention versus the control arm. For example, the following generalized linear mixed model will compute the adjusted odds ratio for taking up a PP implant: *Y*_*ijl*_ = *β*0 + *β*1 × (intervention_*ij*_) + *β*2 × (covariates_*ij*_) + *β*3 × (intervention_*ij*_ × covariates_*ij*_) + *β4*_*j*_ + *u*_*i*_ + *ε*, where *i* = clusters*, j* = time periods, *l* = individuals, *β*1 = fixed effect for intervention, *β*2 = fixed effects for covariates, *β*3 = fixed effects for interactions, *β*4_*j*_ = fixed effect for time, *u*_*i*_ = random effect for ICC, and *ε*_*ijl*_ = random residual. Covariates assessed as potential confounders will include facility volume, service volume-to-staffing ratio, and number of promotions received. Outcomes will be reported as adjusted odds ratios with 95% confidence intervals. We hypothesize that the intervention will significantly increase PP LARC uptake by comparing intervention versus control. We will also assess the independent effect of each promotional strategy on PP LARC uptake. Every promotion will be recorded and we will estimate the independent effect of receiving CHW counseling, video-based promotions, or Happy Client promotions (treating each strategy as an independent, dichotomous exposure of interest) on PPFP uptake using adjusted multivariate regression modeling with general estimating equations methods.

### Interim analyses {21b}

Interim analysis of study (S) AEs will be conducted at the end of year 2. The difference between the proportion of events (the test statistic) in the intervention and the expected rate of (S) AEs associated with each contraceptive method (from the published literature) will be quantified with *p*-values constructed for alpha = 0.05, two-sided. If the test statistic exceeds the boundary, then the study could be considered for early termination. In the event of any adverse or life-threatening events occurring at an unacceptable level, the PI will take appropriate action to halt the study, release a participant from the study, or modify the study procedures to reduce or eliminate the identified risk. Should a situation arise of insufficient power due to poor recruitment or early detection of (S) AEs, the investigators will discuss halting the study, when and if needed, and would report such action to the Emory and Rwandan IRB, the Data and Monitoring Safety Board (DSMB), and the NIH project officer. Completion rates of intervention activities will be monitored in a parallel fashion.

### Methods for additional analyses {20b}

#### Qualitative analyses

We will analyze the checklist quantitatively to identify organizational readiness and common service delivery or facility-level gaps. All in-depth interviews will be audio-recorded, transcribed, and undergo standard thematic analytic coding data analyses [26]. Thematic analysis is the process of identifying patterns within qualitative data without a particular theoretical perspective making it a flexible method. Two trained coders will independently read transcripts and create a preliminary codebook with definitions and examples of processes applicable to each code. Topics described in the interviews will be organized according to anticipated themes. A saturation grid will be used for the analysis of themes. To enhance reliability, coders will compare codebooks and additional themes that emerge will be included after consensus within the research team. Saturation of themes is achieved once insights begin to repeat themselves in the grid [27]. While no established standard for the sample size to achieve saturation exists for in-depth interviews, the number of interviews exceeds expert recommendations [28]. Thus, we assume a priori that we will reach saturation. Coding will be done in the NVivo software (QSR International).

#### Descriptive analyses

Descriptive analyses will evaluate the outcomes of PPFP uptake (by method type), follow-up attendance, side effects (by method type), and client satisfaction. We will assess the association between PPFP uptake and timing of counseling (chi-square test), presence of a male partner (chi-square test), and the number of counseling sessions received (chi-square test for trend).

#### Intervention cost-effectiveness and cost-utility

We will employ standard methods of cost analyses as recommended by the Second U.S. Panel on Cost-effectiveness in Health and Medicine to conduct an economic evaluation from the societal perspective (the health care sector costs plus the costs to the participant for participating in the program) to estimate the cost and cost-effectiveness of the intervention relative to control.

#### National Costed Implementation Plan

We will create a Costed Implementation Plan following the Family Planning 2020 Costed Implementation Plan Resource Kit (https://www.familyplanning2020.org/cip). Our team previously developed National Costed Implementation Plans for Couples’ HIV testing for Zambia and Botswana at the request of their respective MOHs, and the PPFP Costed Implementation Plan will be disseminated to the Rwandan MOH.

### Methods in analysis to handle protocol non-adherence and any statistical methods to handle missing data {20c}

#### Missing data

Given the uncertainty associated with imputing data, primary analyses will be complete case analyses (i.e., missing data will not be replaced). Sensitivity analyses using multiple imputation will evaluate the impact of missing data on results using the fully conditional method in SAS.

#### Crossover effects

The potential for crossover is possible if the nursing staff are redistributed during the study. The MOH will limit redistribution in study facilities during the intervention to only what is necessary (e.g., due to staff shortages). Additionally, it is possible that women could receive promotions in an intervention study facility and then deliver in a control study facility where they request PPFP. We will attempt to limit such events by encouraging women to consistently receive ANC, L&D, and IV services at the same clinic (generally beneficial to women since medical records are not easily linked across facilities). Transfers between intervention and control facilities will be recorded to quantify the potential effect of crossover.

### Plans to give access to the full protocol, participant-level data, and statistical code {31c}

The de-identified participant-level dataset and statistical code may be available upon request to the study team after completion of the study.

## Oversight and monitoring

### Composition of the coordinating center and trial steering committee {5d}

Trial coordination will be led by the PI and co-Is who will meet monthly. The data management team will be led by co-I Nizam and three Emory employees, including the study PI, and this group will meet at least quarterly. Day-to-day organizational support will be provided by the PSF/CFHR staff including two medical doctors and senior nurse counselors who will meet weekly. Emory University facility and resources will be used to support the study to ensure that the study has the administrative, data coordinating, and technical support needed to implement the study aims.

### Composition of the data monitoring committee, its role, and reporting structure {21a}

We will assemble a DSMB consisting of experts in PPFP implementation, policy, and family planning/reproductive health. DSMB membership will be reviewed and approved by the NIH. Should there be any questions regarding the independence of the DSMB they will be addressed and corrected if necessary.

None of the members of the DSMB will have any conflicts of interest (COI) that they need to disclose. Monitoring body members should have no direct involvement with the study investigators or intervention. Each member will sign a COI statement which includes current affiliations, if any, with any steering committees or advisory councils associated with the study, pharmaceutical and biotechnology companies (e.g., stockholder, consultant), and any other relationship that could be perceived as a conflict of interest related to the study and/or associated with commercial or non-commercial interests pertinent to study objectives.

The DSMB will act in an advisory capacity to the NIH to monitor participant safety, evaluate the progress of the study, and review procedures for maintaining the confidentiality of data, the quality of data collection, management, and analyses. The DSMB will meet in-person/Zoom conference at the outset of the study, and thereafter by conference call twice per year scheduled around particular milestones of the study, e.g., prior to study initiation (for review of surveys, materials, protocols, etc.), approximately halfway through recruitment (to ensure adequate recruitment, review progress, protocol fidelity, etc.), at the conclusion of intervention delivery, and a session for the final results. Safety reports are sent to the DSMB twice a year and will include a detailed analysis of study progress, data, and safety issues. Specific triggers for an ad hoc review or the initiation of the process of an ad hoc review will occur if there are unforeseen deaths or the threshold for SAE has been met.

We will have a DSMB charter finalized in parallel to the final study protocol. The charter will detail DSMB’s responsibilities, including the following:

- In year 1, reviewing the research protocol, Data and Safety Monitoring Plan (DSMP), and informed consent documents, including all proposed revisions, tools for data collection, and the manual of operating procedures (MOOP)
- Every 6 months:
  - Evaluating the progress of the study including assessments of data quality, participant recruitment, accrual and retention, participant risk versus benefit, performance of study site(s), and other factors that can affect the outcome
  - Evaluating proposed new sites
  - Considering the impact of factors external to the study when new information/developments become available that may affect the safety of participants, their willingness to participate in the study, or ethics and conduct of the study
  - Reviewing unanticipated problems and serious adverse event reports and inform the NIH whether there is an effect on participant safety
  - Reporting any problems with study conduct or performance to the NIH
  - Ensuring the measures to ensure the confidentiality of study data and results are appropriate
  - Reviewing and evaluating requests for protocol modifications/amendments
  - Reviewing the interim analyses and/or accumulating data at the specified interval(s), and as appropriate and make a recommendation to continue, terminate, or modify the study based on observed benefit or harm in accordance with the planned stopping rules

### Adverse event reporting and harms {22}

Though contraceptive methods have extremely favorable safety profiles, all contraceptives have side effects.

- Copper IUD: Non-serious side effects include pelvic pain (12%), heavy menstrual bleeding (5%), ovarian cysts (4%), and dysmenorrhea (3%). These side effects tend to be worse for the first 3–6 months after insertion and then improve. Serious side effects include infection (5%), perforation of the uterine wall (occurring in 1.6% of women), pelvic inflammatory disease (PID) (occurring in 0.5% of women and typically during the first 3 weeks after insertion), and menorrhagia (i.e., heavy bleeding requiring treatment, 0.2%).
- Hormonal methods (implant, oral contraception, injectables): Common non-serious side-effects include weight gain (46%), headaches (12%), irregular or painful periods (8%), acne (5%), and nausea (4%). Serious side effects include blood clots (< 1%). Insertion site infections may occur with implant insertion (0.8%).
- Tubal ligation: Non-serious side effects include minor bleeding (5%). Serious side effects include severe bleeding (0.5%), severe pain (0.4%), and infection (1%).

All contraceptive methods offered during this program are approved for use in Rwanda. Implementation clinic facility staff will be trained to collect AEs and SAEs from patients for recording within pre-created government clinic logbooks (which detail the incident, actions taken, supervisor notes, and follow-up steps). Any (serious) adverse events following participation will be tracked via referral and clinician follow-up. Events will also be recorded on a study adverse event log. These data are later abstracted by our research staff for electronic data entry.

We will closely track incoming numbers of participants via ongoing recruitment activities, and weekly and monthly reports will be compiled by the study staff. We will utilize similar procedures to monitor any missing data or missed follow-up assessments. (S) AE data reports will be monitored weekly by the study coordinator, the PI, and the study statistician. All data will be protected on firewall-protected servers at Emory and the Rwanda coordinating site. Any identification of recruitment rates or follow-up rates that represent threats to our ability to sufficiently answer the research questions will be met with action by the PI to intervene to remedy the shortcomings.

The study adverse event log, supplemented by any additional staff notes will be provided to the appropriate agencies, including the Rwandan and Emory IRBs and NIH. Any resulting recommendations from the IRB will be communicated to the NIH. The PI will be responsible for daily monitoring and reporting of any adverse events and will involve the DSMB. The study statistician prepares reports that list adverse events, serious adverse events, deaths, and disease- or treatment-specific events required for monitoring body review in order to ensure good clinical care and identify any emerging trends. PI Wall will follow the Emory and Rwandan IRB and Office for Human Research Protections (OHRP) reporting requirements.

Any serious incidents involving subject safety will be reported to all institutional IRBs by the PI within 7 working days. The following information will be included in any report: (1) all serious adverse events associated with study procedures and/or (2) any events or problems involving the conduct of the study or patient participation, including problems with the recruitment or consent processes. The PI will also provide a description of any problems or issues observed during the study to each IRB on an annual basis.

Standard operating procedures (SOPs) will be developed and used to manage possible side effects and adverse events including the following:

- Nurse/counselors will be trained to counsel participants about the possible side effects of PPFP methods. This includes informing women as to the seriousness and likelihood of the side effects and adverse events listed above.
- As part of the SOPs, counselors and nurses will provide follow-up consultations. Women will be asked to self-report side effects including signs of infection, and for PPIUD users, IUD string placement will be checked via bimanual pelvic exam. IUD strings will be trimmed as needed and a pelvic ultrasound given if the strings are not visible during physical exam. Women may decide to discontinue or request a new family planning method of their choice.
- In the first month of intervention implementation at each facility, the study coordinator Dr. Rosine Ingabire and two national PPFP trainers involved in our pilot will have daily supervisory contact with the providers to troubleshoot any study or clinical questions; this support will be phased into an “on call” system for the team to address questions as they arise throughout the trial. We will consult co-I Haddad and local ObGyns as needed for clinical consults. Serious side effects (perforation, PID, and menorrhagia) will result in a referral for specialist care, as is the current standard of care.
- We will adapt case report forms from our previous experience with family planning trials and record all clinical queries, responses from the investigative team, and develop subsequent management plans.

### Frequency and plans for auditing trial conduct {23}

The project management team (comprising key study investigators, including the data management team leadership) will meet weekly to review the trial conduct and progress. The DSMB will meet twice a year to review the safety reports with ad hoc reviews triggered per the charter. Emory and Rwandan ethics committees will review trial conduct annually. Milestone reporting to the National Institutes of Health is due twice yearly along with an annual Research Performance Progress Report (RPPR). SAEs will be promptly reported to the NIH, reporting to the National Institutes of Health, Emory and Rwandan ethics committees, and the DSMB per establish protocols.

### Plans for communicating important protocol amendments to relevant parties {25}

Important protocol modifications will be communicated to investigators, IRBs, trial registries, journals, and regulators via email correspondence from the study PI.

## Dissemination plans {31a}

The following are the dissemination plans:

1. We will register our study in ClinicalTrials.gov within the 3 months of study funding.
2. We will submit the results of the clinical trial to ClinicalTrials.gov within 12 months after the primary completion of the study.
3. Informed consent documents for the clinical trial will include a specific statement relating to posting of clinical trial information at ClinicalTrials.gov.
4. Emory University and PSF/CFHR have internal policies in place to ensure that clinical trials registration and results reporting occur in compliance with policy requirements.
5. Our research will be rapidly disseminated to inform policy changes, future interventions, and NIH proposals in a timely and effective manner. We will work closely with implementing partners including the Rwandan MOH to disseminate our findings.
6. We will publish four or more manuscripts on the outcomes of our research in peer-reviewed journals.

## Discussion

We expect that intervention facilities can serve as training centers for PPFP expansion to the 13 additional facilities in Kigali which offer family planning or L&D services as well as rural areas. We will ensure that systems are in place to expand training activities to cover an even larger proportion of the population. Our PPFP implementation and cost-effectiveness model are designed to be flexible and expandable to other countries in the region, which also have a high unmet need for PPFP. We expect our work to lead to the creation of a toolkit to guide PPFP service implementation. This toolkit will provide a flexible, evidence-based package including best practices for demand creation and modeling/Costed Implementation Plan tools that can be parameterized with facility- and local-specific data (e.g., local salaries) to determine scenarios under which the PPFP intervention is effective and cost-effective in a range of settings. Our team has experience developing and adapting such toolkits for intervention implementation. Our work has the potential to produce an implementation model to increase birth spacing and drastically improve maternal, newborn, and family health across Africa.

## Trial status

Protocol version number and date: 12Feb21 v1.0

Recruitment for PPFP service delivery will begin in March 2022 and end in September 2025.

## Data Availability

All study investigators will have access to the study database. Researchers who provide a methodologically sound proposal and whose proposed use of the data has been approved by the Rwanda National Ethics Committee, to achieve the aims in the approved proposal, may also have access to the data. To gain access, data requestors will need to sign a data access agreement.

## Abbreviations

AE: Adverse event
ANC: Antenatal care
CFIR: Consolidated Framework for Implementation Research
CHFR: Center for Family Health Research
CHW: Community health worker
DHS: Demographic Health Survey
DSMB: Data safety monitoring board
IRB: Institutional Review Board
IUD: Intrauterine device
IV: Infant vaccination
L&D: Labor and delivery
LARC: Long-acting reversible contraception
MOH: Ministry of Health
MOOP: Manual of operating procedures
OHRP: Office for Human Research Protections
PBF: Performance-based financing
PI: Principal investigator
PID: Pelvic inflammatory disease
PP: Post-partum
PPFP: Postpartum family planning
PPIUD: Postpartum intrauterine device
PPLARC: Postpartum long-acting reversible contraception
PSF: Projet San Francisco
SAE: Severe adverse event
SOP: Standard operating procedures
